# Cathepsin K inhibition by VBX1000 alleviates canine periodontitis

**DOI:** 10.3389/fvets.2025.1656782

**Published:** 2025-11-21

**Authors:** Jerzy Gawor, Daria Ziemann Gawor, Isabelle Druet, Julien Salindre, Alicja Gajosz, Paweł Kowalczyk, Noémie Dam, Dominique Tierny, Matthieu Dubruque, Rémy Hanf

**Affiliations:** 1 Klinika Weterynaryjna Arka, Krakow, Poland; 2 Alliance, Bordeaux, France; 3 Clinique Vétérinaire VPLUS, Mirambeau, France; 4 Veterinary Clinic KoniczynaVET, Warsaw, Poland; 5 Braci Mniejszych Veterinary Clinic, Konstantynow Lodzki, Poland; 6 Vetbiolix, Loos, France; 7 OCRvet dba Clinaxel, Lille, France

**Keywords:** cathepsin K, canine periodontitis, open-label study, safety, efficacy, VBX-1000

## Abstract

**Introduction:**

The efficacy and safety of a novel cathepsin K inhibitor, VBX1000, were evaluated in client-owned dogs suffering from periodontal disease.

**Methods:**

This open-label study recruited twenty dogs (*n* = 20) with at least 3 teeth at stage 2 or 3 of periodontal disease. Dogs were orally treated once-a-day with VBX1000 (25 mg/kg or 50 mg/kg, *n* = 10 per group) for 60 days, and then with 50 mg/kg once-a-day for an additional 30 days. The first objective was to assess evolution compared to pre-treatment of plasma carboxy-terminal telopeptide of collagen type 1 (CTX1) used as a marker of target engagement and bone resorption. In each evaluated tooth (*n* = 60; three teeth per dog), evolution counter to baseline of clinical attachment loss (CAL), periodontal probing depth (PPD), and bleeding on probing index (BPI) were evaluated The effects of cathepsin K inhibitor on alveolar bone defects were assessed with intraoral dental radiography (DR) performed at inclusion and at the end of the treatment period. A confirmatory analysis was performed in a subpopulation (*n* = 10 dogs; 30 teeth) using the cone beam computed tomography scan (CBCT) imaging technique.

**Results:**

Throughout the treatment period, VBX1000 was well tolerated. At Day60, plasma CTX1 was significantly and similarly reduced compared with baseline (*p* < 0.05) in the two groups. At the end of the treatment period (at Day90) in the total population (*n* = 20), plasma CTX1 was 0.10 ± 0.04 ng/mL relative to 0.26 ± 0.20 ng/mL at baseline (*p* < 0.001). DR before and after treatment showed decreases in width (*n* = 60 teeth; three teeth/dog; *p* < 0.0001), depth (*p* < 0.05), and height of bone defects measured between the root and the maxillary bone. These effects on bone defects were confirmed in a subpopulation analyzed by CBCT. At the end of the treatment period, clinical attachment loss (CAL) was reduced relative to pretreatment: 2.87 ± 1.73 mm compared to 3.86 ± 2.06 mm (*n* = 60; *p* < 0.0001). Likewise, the periodontal probing depth (PPD) was reduced 2.71 ± 1.03 mm compared to 3.69 ± 1.23 mm (*n* = 60, *p* < 0.0001).

**Conclusion:**

The findings support the inhibition of cathepsin K by VBX1000 as a new therapeutic approach for mild-to-moderate periodontal disease in dogs. A randomized, double blinded placebo-controlled trial in dogs should confirm the potential of VBX1000 in this indication.

## 1 Introduction

Periodontal disease (PD) is the most widespread oral disease in dogs, with studies showing that 60–97% of dogs have some degree of PD (1–4). PD is defined as plaque-induced immune-mediated damage to the periodontium, which anchors the tooth in the maxillary and mandibular bones. If left untreated, PD becomes a slowly evolving chronic inflammatory disease that first affects the gingiva (gingivitis) and then propagates to deeper structures of the periodontium, leading to progressive tooth attachment loss due to damage to the periodontal ligaments and loss of alveolar bone (periodontitis). Based on clinical attachment level and radiographic measurements of the alveolar pocket, the American Animal Hospital Association defines four degrees of PD severity according to stage of periodontitis as follows: gingivitis without periodontitis (PD1), early periodontitis (PD2), moderate periodontitis (PD3), and advanced periodontitis (PD4) (5).

PD is thought to result from complex interactions among plaque bacteria (biofilm), host immune response, and periodontal structure. The initial manifestation is gingival inflammation, which involves the recruitment of many neutrophils to the plaque site and associated release of bacterial toxins, destructive enzymes, and cytokines (6). The first outward clinical sign of gingivitis is erythema, typically in the area adjacent to dental deposits (plaque and calculus), which is followed by edema and halitosis (7, 8). As the inflammation descends deeper into the surrounding periodontal tissues, inflammation-induced bone resorption leads to alveolar bone loss, which is currently irreversible without advanced regenerative surgery (8, 9).

The control or prevention of dental plaque formation and its removal can be achieved by a combination of tooth brushing, special diet, chew toys, and regular professional periodontal treatment (10, 11). Once PD occurs, scaling and root planing can be further accompanied by the administration of antibiotics and nonsteroidal anti-inflammatory drugs (NAIDS) to promote the reduction of gingival inflammation and bleeding, pocket depths, subgingival bacteria, and clinical attachment loss (CAL) (10).

There are no pharmacological treatments or drugs able to directly suppress the alveolar bone resorption process and promote alveolar bone regeneration in dogs with periodontitis. Therefore, surgical techniques, including osseous grafting, guided tissue regeneration, and the use of bioactive products (such as bone morphogenic protein) (10, 12–15), are proposed for treating the most severe cases of PD (PD3 and PD4). When periodontal regeneration techniques and associated dental hygiene care are not achievable due to high surgery costs, tooth extraction is necessary (11, 16, 17).

In humans, cathepsin K is the major enzyme involved in the bone resorption process (18). Cathepsin K is predominantly secreted by activated osteoclasts to degrade type I collagen, osteonectin, and other bone matrix proteins during the bone resorption process (19). Inappropriate upregulation of cathepsin K has been suggested in some bone disorders, including osteoporosis (19) or PD (20–30). Given its elevated levels in chondroclasts of the osteoarthritic synovium, cathepsin K is also implicated in rheumatoid arthritis in human patients (31). As a result, cathepsin K inhibitors have been a subject of extensive research during the last decades (32). A phase three trial has shown that long-term treatment with a cathepsin K inhibitor, namely, odanacatib, significantly reduces osteoporosis and the risk of bone fracture in postmenopausal woman (33). However, further development of odanacatib has been discontinued, and no cathepsin K inhibitor has received regulatory approval in either human or animal healthcare to date.

VBX1000 (previously named MIV701) is a new, highly selective cathepsin K inhibitor that is not structurally related to odanacatib. Previous tolerance studies have been performed in rats, dogs, and monkeys, revealing no major safety concerns in the cardiovascular system. VBX1000 is currently in development to obtain regulatory approval as the first veterinary treatment of PD in companion animals. This publication presents the results of the first proof-of-concept (PoC) clinical study assessing the efficacy and safety of a cathepsin K inhibitor in client-owned dogs with spontaneously developed mild to moderate PD. The results provide initial evidence of the efficacy of once-a-day oral administration of VBX1000 for 90 days against major clinical manifestations of PD, supporting the regenerative action of VBX1000 against alveolar bone loss. With the safety profile observed throughout the treatment period, these results support further development of VBX1000 in this indication.

## 2 Materials and methods

### 2.1 Study design and objectives

The aim of the open, multicenter, European VBX1200-CL-1001 PoC clinical study (5 sites in France and Poland) was to evaluate the safety and efficacy of repeated oral administration of VBX-1000-besylate salt (VBX1000) for 90 days for reduction of plasma carboxy-terminal telopeptide of collagen type 1 (CTX1) levels and alleviation of various PD symptoms in dogs. The PoC study aimed to include a minimum of 20 dogs with mild to moderate PD (stage 2–3) affecting at least three teeth.

Five visits were scheduled for each dog as follows: a screening visit 0–7 days before the inclusion visit at Day 0; two intermediate visits after 30 days (Day 30) and 60 days (Day 60) of treatment for safety follow-up with blood sampling; and an end-of-study visit after 90 days of treatment (Day 90).

At the inclusion visit at Day 0, after performing professional dental scaling and polishing followed by thorough clinical and radiographic examination, three teeth with stage 2–3 PD were selected. After initial scaling and polishing of teeth, canine patients that met the inclusion criteria were randomly assigned to two parallel groups of treatment in a 1:1 ratio. Group 1 was treated with VBX1000 at a daily oral dose of 25 mg/kg for 60 days. If at the Day 60 intermediate visit, the dose of 25 mg/kg/day was well tolerated by the patient, a daily dose of 50 mg/kg of VBX1000 was then administered for the following 30 days up to Day 90. Group 2 was treated with VBX1000 at a daily oral dose of 50 mg/kg orally for 90 days (from Day 0 to Day 90).

The primary objective of the study was to investigate the effects of VBX1000-besylate salt (VBX1000) on plasma CTX1 concentration after 90 days of treatment. Plasma CTX1 is a marker of the bone resorption process and cathepsin-K activity (32). The secondary objectives of the study focused on the changes in the following clinical parameters that characterize PD in 3 selected teeth in 20 dogs after 90 days of treatment: CAL, periodontal probing depth (PPD), and bleeding-on-probing index (BPI). Additionally, the radiographic changes of the alveolar bone defects after 90 days were assessed on the same teeth using two imaging techniques intraoral dental radiography (DR) for all patients and cone beam computed tomography (CBCT) scan in 10 dogs.

### 2.2 Inclusion and exclusion criteria

The owners of participating dogs agreed with study conditions and signed the applicable consent forms. Female dogs known to be pregnant or lactating were excluded. To be eligible, dogs were required to be adult mesocephalic or dolichocephalic breeds (brachycephalic breeds were excluded) with permanent dentition and having at least 3 teeth with mild (stage 2) to moderate (stage 3) PD according to the staging system of the American Animal Hospital Association (5). All animals were fed a dry diet and received water ad libitum during the entire research period. None of the dogs received passive or active oral homecare, such as tooth brushing or dental diet, before and after the study. In addition to the presence of PD, dogs were otherwise considered healthy by the investigator. Moreover, dogs with gingival hyperplasia, neoplasia, autoimmune disease, immunosuppressive disease, chronic systemic disease or medical conditions requiring long-term treatment with non-steroidal anti-inflammatory drugs (NSAIDs) or steroids were excluded. To be eligible, dogs must not have received treatment with NSAIDs within the last two weeks before the inclusion visit. Similarly, treatment with short-, intermediate-, or long-acting steroids within the last 2 weeks, 4 weeks, and 12 weeks before the inclusion visit, respectively, was considered an exclusion criterion. Dogs receiving chronic treatment with bisphosphonates were also excluded.

### 2.3 Concomitant treatments

During the treatment period (from Day 0 to Day 90), any medication, particularly treatment with steroids and bisphosphonates, which could possibly interfere with the results of the study was not permitted. If considered necessary by the investigator at the intermediate visit or at any unscheduled visit, for whatever medical reason, short-term treatment with NSAIDs was allowed for a maximum of 3 consecutive days, and this duration could be extended to 5 days in case of surgical treatment at intermediate visits.

### 2.4 Doses and drug administration

The VBX1000 doses were selected based on a previous pharmacokinetic and tolerance study performed in healthy beagle dogs, which indicated that once-a-day repeated oral administration of VBX1000 at 1 mg/kg, 10 mg/kg and 100 mg/kg in healthy beagle dogs for 14 days is well tolerated up to the highest dose tested (VBX1000 preclinical dossier). At 10 mg/kg/day and 100 mg/kg/day, VBX1000 shows cathepsin K engagement by decreasing the level of CTX1. For the present PoC study, the 25 and 50 mg/kg/day doses were selected to assure both target engagement and tolerance in dogs with PD.

VBX1000 (1 to 3 capsules per day depending on body weight) was administrated to the dog by the owner once per day at home with a standard meal. The capsule(s) could be administered with a small amount of wet food or favorite snack, but the capsule could not be opened and poured over the food.

### 2.5 Primary and secondary endpoints

The plasma CTX1 concentration was measured in all dogs at the inclusion visit (Day 0) and at the follow-up visits (Day 30, Day 60, and Day 90). The primary objective of the present study was to investigate the effects of VBX1000-besylate salt (VBX1000) on plasma CTX1 concentration after 90 days of treatment. Plasma CTX1 is a marker of the bone resorption process and cathepsin-K activity (34). The secondary objectives of the present study focused on the changes in the following clinical parameters that characterize PD in 3 selected teeth in 20 dogs after 90 days of treatment: CAL, PPD, and BPI. Additionally, the radiographic changes of the alveolar bone defects after 90 days were assessed on the same teeth using two distinct imaging techniques—DR for all patients and cone beam computed tomography (CBCT) scan in 10 dogs. In total, 30 teeth in 10 dogs (6 maxillary and 24 mandibular teeth) received both diagnostic modalities.

The dose-dependent effects of VBX1000 on plasma CTX1 were evaluated using blood samples collected at intermediate visits on Days 30 and 60.

The effects of VBX1000 treatment on secondary endpoints, including changes (Day 90 compared to Day 0) in PD parameters (CAL, PPD, and BPI) and alveolar bone loss dimensions (height, depth, and width of alveolar bone loss), were measured via probing and intraoral radiographs and CBCT scans, respectively, on the 3 teeth per dog selected at the inclusion visit (Day 0).

### 2.6 Statistical analysis

Statistical analyses were performed using GraphPad-PRISM^®^ software (Boston MA, United States). The primary endpoint involved comparing the plasma CTX1 levels at the end of the treatment period to those at baseline (Day 90 compared with Day 0) in each group (*n* = 10 per group) and in the total population (*n* = 20). For this analysis, each dog was its own control. In each group, because the plasma CTX1 concentrations at baseline were not normally distributed according to Shapiro–Wilk test, the statistical analysis on the primary endpoint was performed without normality assumption using Wilcoxon matched-pairs signed rank test (non-parametric).

The time-dependent effects of treatment on plasma CTX1 were evaluated by measuring the changes in plasma CTX1 at Days 30 and 60 in each group compared with baseline using a non-parametric Kruskal-Wallis test (analog of analysis of variance [ANOVA] for non-normally distributed variables) followed by Dunn’s test for multiple comparisons. In this non-controlled study, there was no formal intergroup comparison for plasma CTX1 to estimate the effect-size change relative to a comparator. However, the changes in plasma CTX1 levels measured at Day 60 in the two groups were compared via a Mann–Whitney U test.

For assessing the effects of treatment on CAL, PPD, and BIP, as well as on the height, depth, and width of the bone defect, at Day 90 compared with baseline, each dog was its own control. Because the baseline values were not normally distributed for the secondary variables according to Shapiro–Wilk test, the changes between Day 0 and Day 90 were analyzed without normality assumption using Wilcoxon matched-pairs signed rank test (non-parametric test). As complementary statistical analyses, a within group comparison was performed for all parameters, comparing the least squares mean (LS mean) value at Day 90 to the LS mean value at Day 0 using a linear mixed-effect model with the individual dog as a random effect.

### 2.7 Safety

A full clinical examination of every participating dog was performed at each visit, and potential adverse events (AEs) occurring since the previous visit were recorded based on feedback from the owner. At the screening visit, intermediate visits, and at the end-of-treatment visit, blood samples were collected for hematology and clinical biochemistry analyses. The hematology analyses included measurement of the following parameters: white blood cells, lymphocytes, monocytes, polynuclear cells (neutrophils, eosinophils, and basophils), red blood cells (RBCs), hematocrit (HCT) levels, hemoglobin (HGB) levels, reticulocyte count (RETIC), mean corpuscular volume (MCV), mean corpuscular hemoglobin (MCH), mean corpuscular hemoglobin concentration (MCHC), and platelet count (PLT). After centrifugation and preparation of plasma samples, biochemistry analyses were performed, including measurement of the levels of albumin, alanine transaminase (ALT), alkaline phosphatase (ALP), creatinine, globulins, glucose, total proteins, urea, albumin/globulin ratio, and urea/creatinine ratio. All of the routine analyses were performed at onsite laboratories immediately after collection of samples.

### 2.8 CTX1 analysis

For CTX1 analysis, a whole-blood sample was collected in anticoagulation tubes (containing EDTA, BD Vacutainer, United States) at the inclusion visit (Day 0), at the intermediate visits (Day 30 and Day 60), and at the end-of-treatment visit (Day 90). The blood sample (2 mL) was collected between 08:00 and 10:00 in the morning after overnight fasting, approximately 12 h after administration of the last treatment. Plasma samples were immediately stored at −20 °C before shipping to the central laboratory for determination of CTX1 levels (CERBA Xpert, France). Plasma CTX1 levels were measured by an electrochemiluminescence immunoassay (Elecsys *β*-CrossLaps/serum; Roche Diagnostics, UK) according to CERBA laboratory procedure.

### 2.9 General anesthesia

After examination of the oral cavity in conscious patients, general anesthesia was performed at the inclusion visit (Day 0) and at the end-of-treatment visit (Day 90) to allow clinical assessment and measurement of PD parameters, to acquire intraoral radiographs, to perform CBCT scans, and to perform professional dental scaling and polishing. General anesthesia was administered by a team in accordance with the relevant standards of care and standardized protocols.

Sedation was performed using recommended doses of medetomidine (Sedator, Eurovet Animal Health B.V. Germany) and butorphanol (Torbugesic, Zoetis, Polska Sp. z o.o. Poland). Preoxygenation was then performed using a mask for delivery of medical oxygen. After sedation was achieved, animals received propofol (Proposer, Corden Pharma S.p.A. Norway) at recommended doses to induce general anesthesia. An endotracheal tube was placed, and the cuff was filled. Cardio monitor peripheral probes were attached to the animal’s body, and a temperature maintenance system was set up. General anesthesia was maintained using isoflurane (Isospire, Dechra, United States) and oxygen for the duration of the dental procedure.

### 2.10 Dental procedures at day 0

At the inclusion visit (Day 0), the initial oral exam was performed in conscious dogs and a thorough oral examination was performed under general anesthesia. Professional dental scaling and polishing were performed, followed by full mouth radiography or CBCT scan or both. Every investigator followed the 2019 American Animal Hospital Association (AAHA) Dental Care Guideline for Dogs and Cats (5) as well as the World Small Animal Veterinary Association (WSAVA) Global Dental Guidelines (35). In accordance with these documents, the entire dentition was scaled supra-gingivally with the use of an ultrasonic scaler and a hand scaler. Subgingival scaling was then performed using a curette to remove subgingival plaque and calculus. After scaling, the tooth crowns were polished using a disposable low-speed prophy angle handpiece with individually packaged fine grit prophy paste or moistened pumice flour placed on a polishing cup running at no more than 3,000 rpm. After prophylactic procedures, gingival sulcus lavage was completed with the use of saline to remove debris and polishing paste remnants. Air or a water syringe were used to inspect the visible subgingival areas for remaining calculus requiring removal. Only necessary procedures aimed at relieving pain were performed, and other procedures were postponed to the end of studies. No advanced periodontal procedures or techniques were applied at Day 0.

### 2.11 Periodontal parameters measured under general anesthesia

At the inclusion visit (Day 0), a minimum of three teeth with stage 2 or stage 3 PD were selected, and CAL, PPD, and BPI were evaluated on each selected tooth under general anesthesia (36). The same teeth were assessed in the same manner at the end-of-treatment visit (Day 90). For these measurements, a calibrated periodontal probe was applied at three to six sites surrounding each selected tooth. All clinical measurements at Day 0 and Day 90 were performed by the same researcher with experience in periodontology (JG). The mean CAL, PPD, and BPI were then calculated for each selected tooth. CAL was defined as the distance from the cementoenamel junction to the bottom of the periodontal pocket and was expressed in millimeters (mm) and rounded to the nearest lower value. PPD was defined as the distance from the free gingival margin to the bottom of the gingival sulcus and was expressed in millimeters (mm) and rounded to the nearest lower value. BPI was assessed at each site of the PPD and CAL measurements. Depending on the absence or presence of bleeding within 10 s following probing of the tooth, BPI was scored either as zero or one, respectively.

### 2.12 Intraoral dental radiographs and CBCT scans

**Figure 1:**
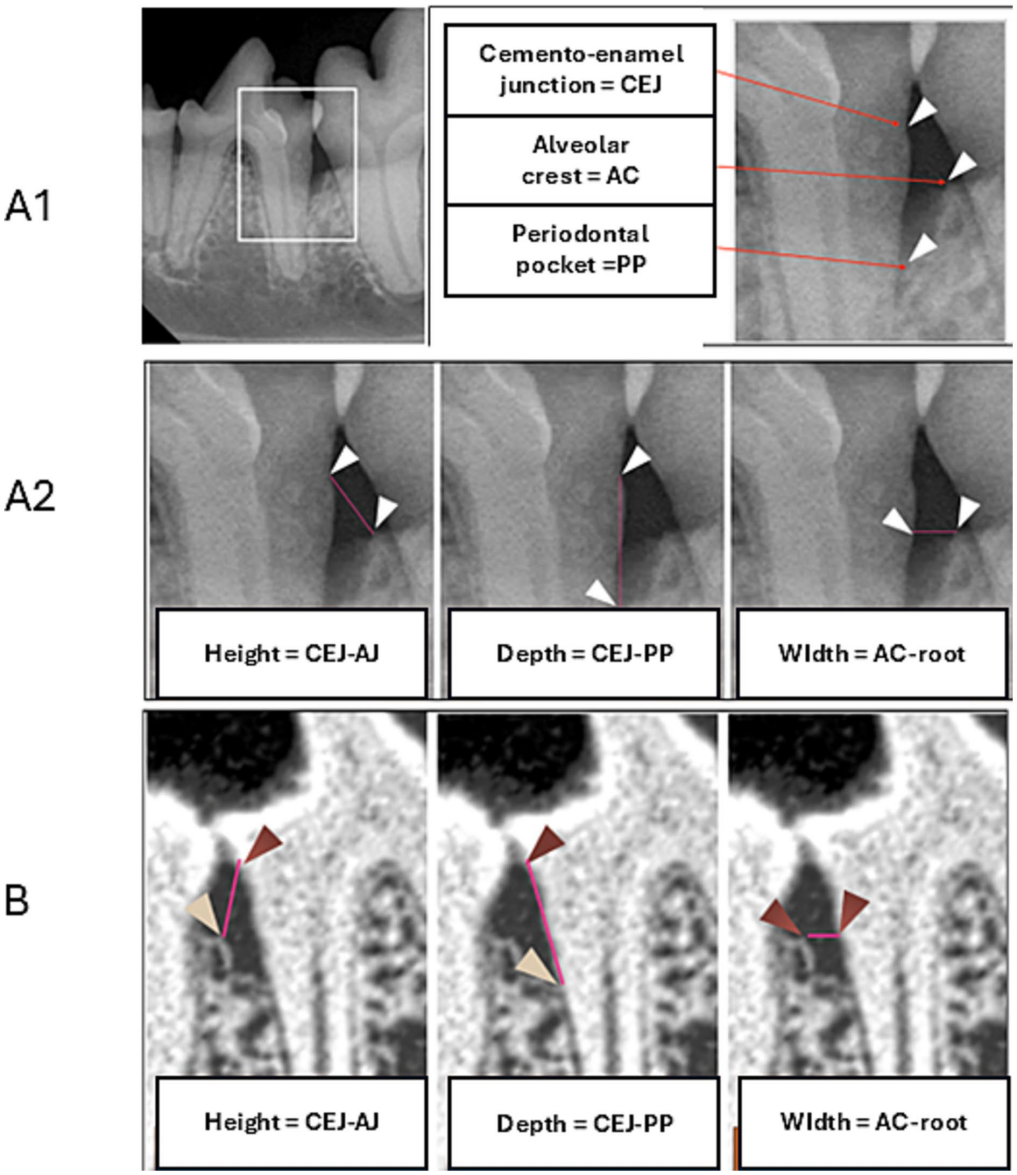
Landmarks for radiographic assessment **(A1)**. Measurements of the alveolar bone defect including height, depth, and width in two-dimensional (2D) imaging **(A2)** and three-dimensional (3D) imaging **(B)**.

Intraoral dental radiographs of each selected tooth were obtained at the inclusion visit (Day 0) and at the end-of-study visit (Day 90) to evaluate the effect of the treatment on the extent of alveolar bone loss. The following distances were measured: height, depth, and width of the alveolar bone defect (37). An example of a periodontal defect with dimensions is shown in Figures 1A1,A2. Among the dimensions, the height of the alveolar crest was defined as the distance from the cementoenamel junction to the upper rim of alveolar crest, the depth of the bone defect was defined as the distance from the cementoenamel junction to the bottom of the bone defect, and the width of the bone defect was defined the distance from the highest point of the alveolar crest to the dental root adjacent to the defect (Figure 1B). These distances were measured on both the mesial and distal sides of the selected teeth using the imaging software digital ruler, which was calibrated each time in the sample plane of the tooth with the use of the UNC 15 calibrated periodontal probe (Vet-Exam Pro Ink 1.2.0. Duerr NDT GmbH Bietigheim-Bissingen, Germany), which is routinely used at the veterinary clinic. The 1 mm calibration was equal to 47,677 pixels, with an expected error maximum of 2%.

In a subpopulation of canine patients recruited at a highly specialized Polish center, 10 of 20 dogs additionally received high-resolution images obtained using full-mouth CBCT (NewTom, 5G XL, Bologna, Italy) performed at the inclusion visit (Day 0) and end-of-study visit (Day 90). CBCT scans were analyzed with the use of NNT viewer software provided by the manufacturer (version: 10.1; QR SRL, Verona, Italy). At both times, volumetric assessment of dentition was set up for the same high-resolution mode (10 × 10 cm with 0.15 mm layers). Both readings were made in the same orientations of axes and locations, measuring alveolar bone loss with the use of maximum magnification (36). To evaluate the effect of the treatment on the alveolar bone loss, the same distances were measured as those described above for the intraoral dental radiographs (Figure 1C).

### 2.13 Ethics

The owners of the participating dogs provided written informed consent for participation in the study. After consideration of risks and benefits, the protocol of the present PoC study was reviewed and approved by the Ethics Committee of the veterinary Clinical Research Organization (OCRvet dba Clinaxel, France) in charge of clinical operations.

## 3 Results

In total, 21 of 24 client-owned dogs screened were enrolled in the present study at five veterinary clinics in France and Poland between December 21, 2022, and July 6, 2023. The patient demographics are presented in Table 1. At one clinical center, almost half of the total study population included dogs from a single Schnauzer breeder, but these dogs were not related to one another and were equally randomized in the two groups of treatment. There was no major imbalance between the two groups of treatment regarding age, body weight, or CTX1 plasma concentration at the inclusion visit. All dogs, except one, completed the study with a full efficacy dataset. Because treatment was discontinued for one dog in Group 1 before the first intermediate visit at Day 30, the treatment value for plasma CTX1 could not be used, and no PD parameters were obtained posttreatment for this canine patient.

**Table 1:** Patient demographics and baseline values.

		Group 1 25 mg/kg	Group 2 50 mg/kg	Group 1 + Group 2
*N*		11	10	21
Breed	Schnauzer	5	5	10
	Pomeranian	1		1
	Mixed	1	1	2
	Italian greyhound		1	1
	Chinese crested dog	1		1
	Dachshund	1	1	2
	Retriever	1		1
	Yorkshire terrier	1	1	2
	Jack Russel		1	1
Sex	Female intact	5	4	9
	Female neutered	3	3	6
	Male intact	2	2	4
	Male neutered	1	1	2
Body weight; kg median (range)		5.7 (3.5–18.8)	7.2 (4.0–10.3)	5.9 (3.5–18.8)
Age; year median (range)		8 (2–11)	6 (4–11)	6 (2–11)
CTX1; ng/mL mean±95% CI		0.27 ± 0.16	0.26 ± 0.112	0.26 ± 0.09

ns denotes non-significant difference (*p* > 0.05) compared with Group 1 using Mann–Whitney U test.

**Figure 2:**
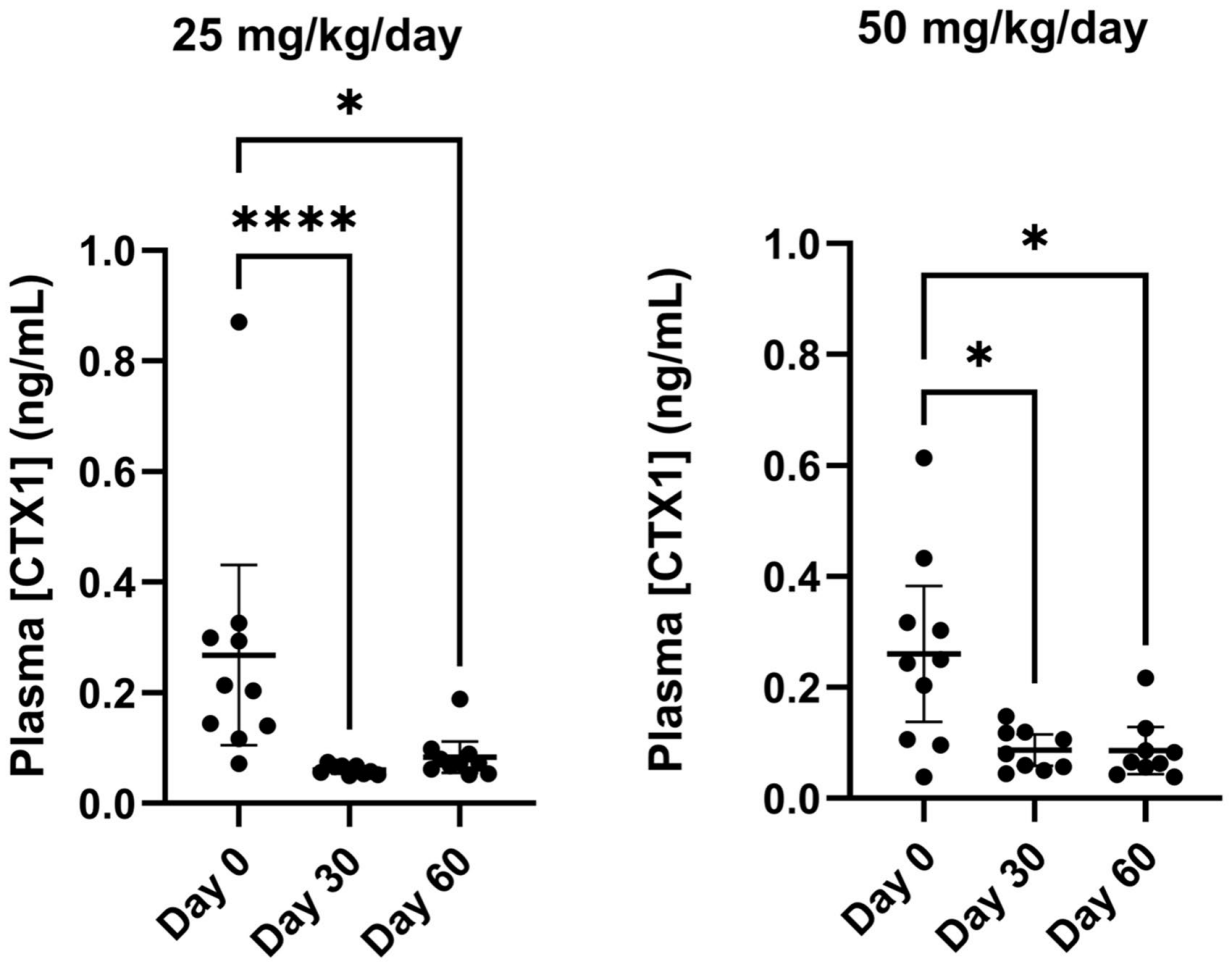
Distribution and change in CTX1 plasma concentration in each treatment group (25 mg/kg/Day left and 50 mg/kg/day right) between Day 0 and Day 60. *p < 0.05 and ****p < 0.001 compared with Day 0 according to Kruskal-Wallis test followed by Dunn’s test for multiple comparisons.

Compared with the baseline value at Day 0, repeated oral administration of VBX1000 at doses of 25 mg/kg/day and 50 mg/kg/day resulted in a significant reduction of plasma CTX1 at Day 30 and Day 60 in both groups, without dose-dependent effects (Figure 2; Table 2).

**Table 2:** CTX1 plasma concentration at days 0, 30, 60 and 90.

		Day 0 pretreatment	Day 30	Day 60	Day 90	*P* value^$^ day 90 compared with Day 0
Group 1	*N* (patients)	10	9	10	10	
	Dose		25 mg/kg/d	25 mg/kg/d	50 mg/kg/d	
	CTX1 (ng/mL)	0.27 ± 0.16	0.06 ± 0.01***	0.08 ± 0.03*	0.10 ± 0.03	*p* = 0.004
Group 2	*N* (patients)	10	9	9	10	
	Dose		50 mg/kg/d	50 mg/kg/d	50 mg/kg/d	
	CTX1 (ng/mL)	0.26 ± 0.12	0.09 ± 0.03*	0.09 ± 0.04*	0.09 ± 0.04	*p* = 0.03
Group 1 + Group 2	*N* (patients)	20	NA	NA	20	
	CTX1 (ng/mL)	0.26 ± 0.09	NA	NA	0.10 ± 0.02	*p* = 0.0002

The plasma CTX1 concentrations are expressed as the mean±95% CI. The *p* value^$^ (Day 90 compared with Day 0) was calculated according to Wilcoxon matched-pairs signed rank test (primary endpoint). The changes in plasma CTX1 concentrations among Days 0, 30, and 60 were analyzed using Kruskal-Wallis test followed by Dunn’s test for multiple comparisons. *p < 0.05 and ***p < 0.001 compared with Day 0. *n* < 10 results from missing values at intermediate visits. NA = not applicable.

Given there was no safety concern in either group at Day 60, all dogs were treated with 50 mg/kg/day for the remaining 30 days of the treatment period (i.e., for dogs in the 25 mg/kg/day group, the dose was increased to 50 mg/kg/day up to Day 90). As shown in Table 2, the primary endpoint of the study was reached in both treatment groups and in the total population, with a statistically significant reduction in plasma CTX1 at Day 90 relative to the pretreatment baseline value at Day 0. The median percent change in plasma CTX1 in the total population (*n* = 20) was −59%.

**Figure 3:**
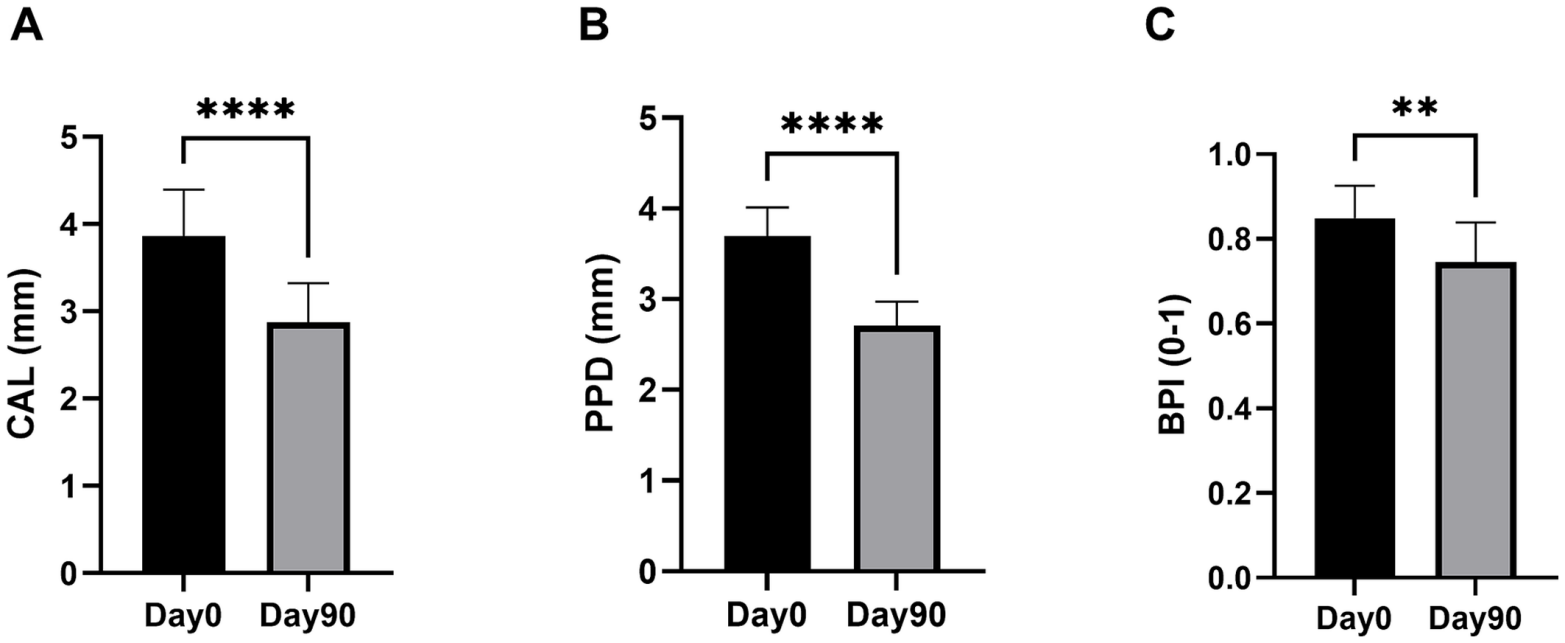
Changes in CAL **(A)**, PPD **(B)**, and BPI **(C)** at the end of the 90-day treatment period compared to Day 0 in teeth of the study population. The data are expressed as the mean±95% CI of mesial + distal measurements on 60 teeth selected in 20 dogs (3 teeth/dog). **p < 0.01 and ****p < 0.001 compared with Day 0 according to Wilcoxon matched-pairs signed rank test.

In each individual dog, CAL, PPD and BPI were measured at Day 0 and Day 90 on 3 teeth with PD, as described in the Methods section. Among 60 evaluated teeth (20 dogs; 3 teeth per dog), the vast majority (92%) were mandibular premolars and molars, and there was no difference in the type of teeth between the two groups (93% in Group 1 and 90% in Group 2; Table 3). There was no significant difference between the two groups when considering baseline CAL (*p* = 0.291), PPD (*p* > 0.999), or BPI (*p* = 0.584) (Table 3). At the end of the treatment period, CAL and PPD were significantly improved relative to the pretreatment value (Day 0) in both groups, and the improvement of BPI was significant only in Group 2 (Table 3). All the canine patients received 50 mg/kg/day at Day 90, and the treatment effects on CAL, PPD, and BIP were comparable in the two groups. The overall effects of VBX1000 on CAL PPD, and BPI on the total number of teeth are presented in Figures 3A–C, respectively. In the entire population (*n* = 20), complementary analyses using linear mixed models with the dog as a random effect confirmed statistically significant changes in CAL (LSmean±95% CI = −0.99 ± 0.40 mm; *p* < 0.001) and PPD (LSmean±95% CI = −0.98 ± 0.34 mm; *p* < 0.001) at Day 90 compared with Day 0 in the study population. Moreover, a generalized linear mixed model with the visit as a fixed effect showed a significant reduction of risk of bleeding according to the BPI risk ratio (Day 90/Day 0) of 0.912 ± 0.06 (*p* = 0.009).

**Table 3:** Type of teeth and changes in PD parameters measured at the end of the treatment period.

		Group 1 (25 mg/kg + 50 mg/kg/day)	Group 2 (50 mg/kg/day + 50 mg/kg/day)	Group 1 + Group 2
Number of teeth (3 per dog)		30	30	60
Type of teeth (*n*)	Incisors	1	3	4
	Canine	1	0	1
	Premolar	15	16	31
	Molar	13	11	24
CAL (mm; mean±95% CI)	Day 0	4.14 ± 0.89	3.58 ± 0.63	3.86 ± 0.53
	Day 90	3.28 ± 0.75	2.47 ± 0.48	2.87 ± 0.45
	*P* value	=0.0001	<0.0001	<0.0001
PPD (mm; mean±95% CI)	Day 0	3.66 ± 0.51	3.72 ± 0.42	3.69 ± 0.32
	Day 90	2.85 ± 0.36	2.56 ± 0.41	2.71 ± 0.27
	*P* value	<0.0001	<0.0001	<0.0001
BPI (mean±95% CI)	Day 0	0.82 ± 0.12	0.88 ± 0.10	0.84 ± 0.08
	Day 90	0.74 ± 0.14	0.75 ± 0.13	0.74 ± 0.09
	*P* value	NS	<0.05	<0.01

The *p* values were calculated according to Wilcoxon matched-pairs signed rank test. ns denotes non-significant difference (*p* > 0.05) compared with group 1 according to the Mann–Whitney U test.

**Figure 4:**
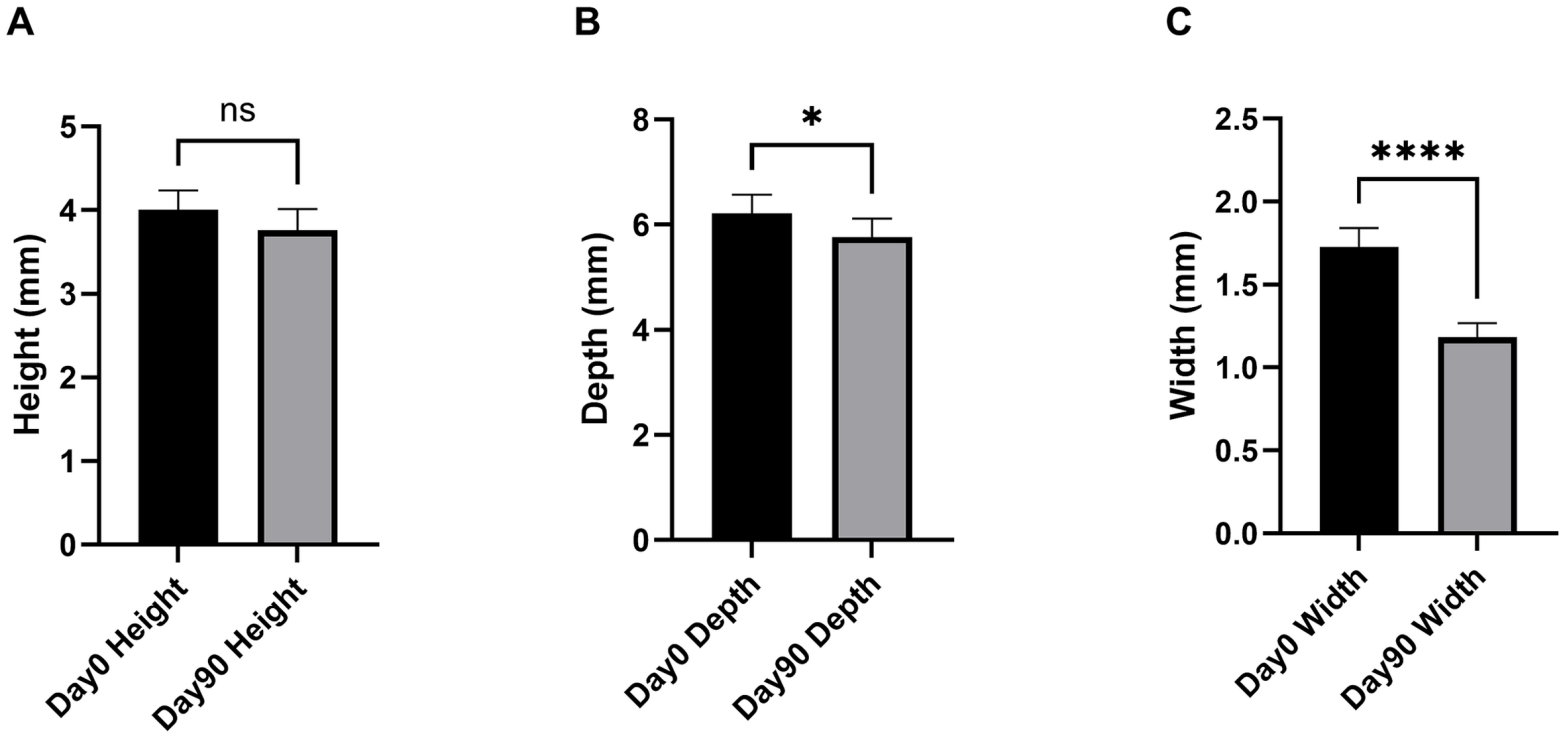
Changes in the height of the alveolar crest height **(A)**, depth of the alveolar bone defect **(B)**, and width of the alveolar bone defect **(C)** measured via DR performed at Day 0 and Day 90. The data are expressed as the mean±95% CI of mesial + distal measurements on 60 teeth selected in 20 dogs (3 teeth/dog). *p < 0.05 and ****p < 0.001 compared with Day 0 according to Wilcoxon matched-pairs signed rank test.

**Figure 5:**
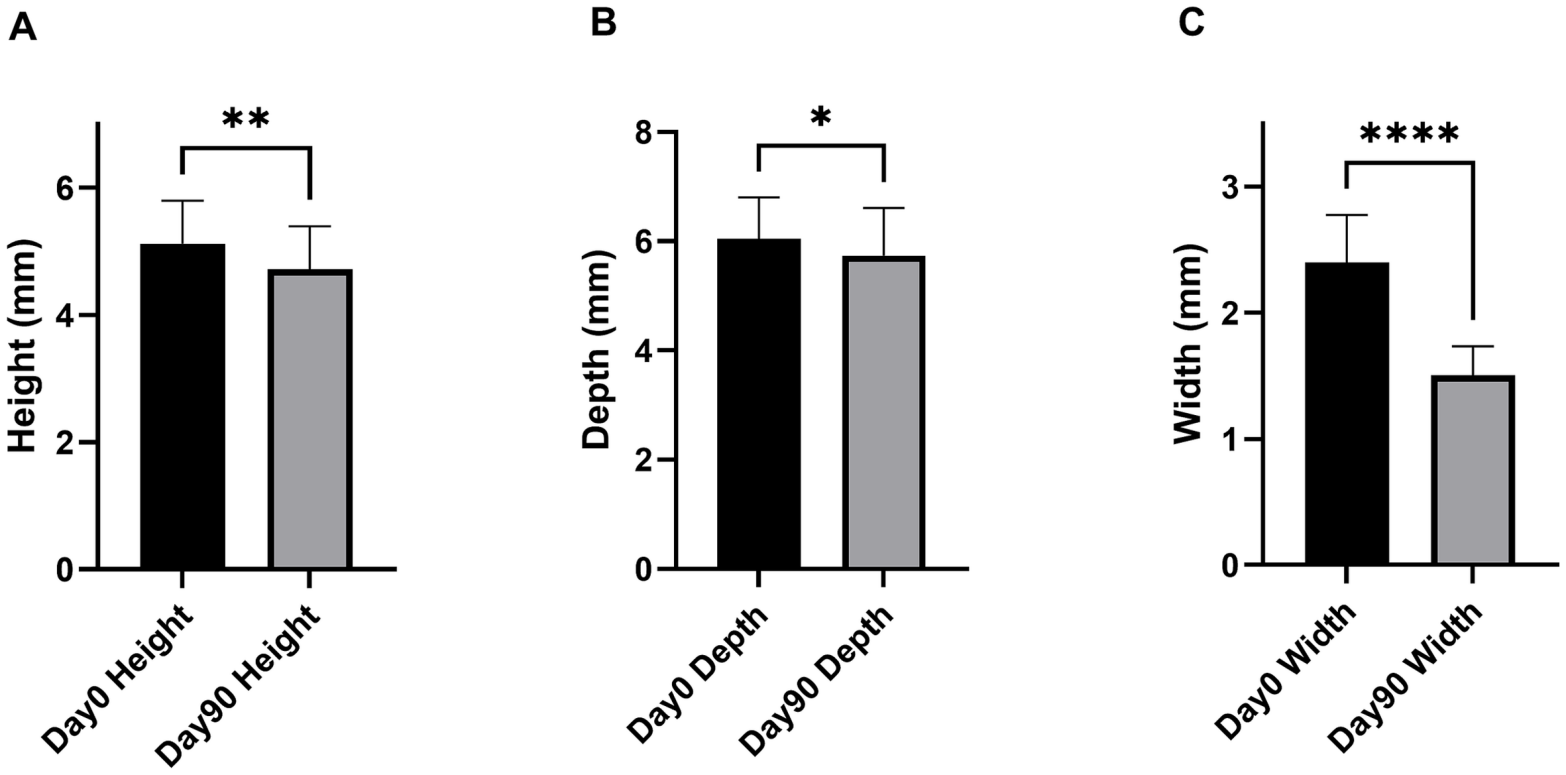
Change in the height of the alveolar crest **(A)**, depth of the alveolar bone defect **(B)**, and width of the alveolar bone defect **(C)** measured on CBCT scans performed at Day 0 and Day 90. The data are expressed as the mean±95% CI of mesial + distal measurements on 30 teeth selected in 10 dogs (3 teeth/dog). *p < 0.05, **p < 0.01, and ****p < 0.001 compared with Day 0 according to Wilcoxon matched-pairs signed rank test.

Comparison of DR of 60 teeth (3 teeth per dog) before and after the 90-day treatment period (Day 90 compared to Day 0) in all 20 dogs revealed significant improvements in the depth and width of alveolar bone defects (Figures 4B,C, respectively), while only a non-significant reduction was observed in the height of the alveolar crest (Figure 4A). Complementary analyses using linear mixed models with the dog as a random effect showed reduction in the height of the alveolar crest (LSmean±95% CI = −0.25 ± 0.42 mm; *p* = 0.240), depth of the alveolar bone defect (LSmean±95% CI = -0.46. ± 0.42 mm; *p* = 0.150), and width of the alveolar bone defect (LSmean±95% CI = -0.55. ± 0.44 mm; *p* = 0.0034) at Day 90 compared with Day 0. The DR results were confirmed using CBCT scans in a subpopulation of 10 dogs that were enrolled in a specialized center. As shown in Figure 5, the high-resolution imaging technique revealed significant improvements in both the height of the alveolar crest (Figure 5A) and depth and width of the alveolar bone defect (Figures 5B,C, respectively). In this subpopulation, linear mixed models with the dog as a random effect similarly showed a reduction in the height of the alveolar crest (LSmean±95% CI = −0.40 ± 0.25 mm; *p* = 0.005), depth of the alveolar bone defect (LSmean±95% CI = -0.30. ± 0.44 mm; *p* = 0.146), and width of the alveolar bone defect (LSmean±95% CI = -0.90 ± 0.57 mm; *p* = 0.007) at Day 90 compared with Day 0.

A full clinical examination was performed at each visit to record AEs and collect blood samples. Throughout the study, oral treatment with VBX1000 was well tolerated in the two treatment groups. No treatment-related serious AEs were reported. One dog in Group 1 developed hemorrhagic gastroenteritis, requiring hospitalization 11 days after inclusion. Because treatment with VBX1000 was discontinued at Day 11, this patient was not included in the efficacy dataset. Owing to the complex clinical procedure and multiple concomitant medications (amoxicillin/clavulanic acid and robenacoxib) administered during this period, analysis of the relation to VBX1000 treatment was inconclusive. With regard to the other dogs, transient non-serious gastrointestinal events (diarrhea, constipation, and vomiting/nausea) were observed in each group, which resolved spontaneously within 1–3 days under treatment.

Hematology analyses at Day 30, Day 60 and Day 90 revealed no significant changes in white blood cells, lymphocytes, monocytes, polynuclear cells (neutrophils, eosinophils, and basophils), RBCs, HCT levels, HGB levels; RETICs, MCV, MCH, MCHC, and PLT. Similarly, biochemistry analyses revealed no significant changes in albumin, ALT, ALP, creatinine, globulins, glucose, total proteins, urea, albumin/globulin ratio, and urea/creatinine ratio.

## 4 Discussion

The present PoC study is the first to evaluate the efficacy and safety of an orally available drug candidate for spontaneously developed PD, either in humans or in animals. In client-owned dogs after repeated once-a-day oral administration, the present findings demonstrated that VBX1000 acts on its molecular target, as evidenced by significantly decreased plasma levels of CTX1, a well-known circulating marker of collagen I degradation induced by cathepsin K (34). In addition, the present results suggested that sustained inhibition of cathepsin K activity by VBX1000 for 90 days is associated with a significant regeneration of alveolar bone defect around affected teeth, together with an overall improvement in key clinical features of PD, as evidenced by measuring CAL, PPD, and PBI.

The two doses of VBX1000 tested produced a similar decrease in plasma CTX1 at Day 30 and Day 60, suggesting that the maximal inhibition of cathepsin K activity after oral administration of VBX1000 was reached at the lowest dose of 25 mg/kg/day from the first 30 days of treatment and then remained constant throughout the treatment period with no apparent desensitization. The present study design did not permit precise assessment of the onset of the effect of VBX1000 on plasma CTX1 during the first month of treatment; however, previous pharmacokinetic studies in healthy beagle dogs have shown a close correlation between daily change of VBX1000 plasma concentration and decrease in plasma CTX1, either after single or repeated oral administration (VBX1000 preclinical package). On the basis of these findings, the inhibition of the bone resorption process likely starts immediately after the first administration. Continuous inhibition of cathepsin K throughout the treatment period may be associated with progressive amelioration of PD, leading to clinically significant effects on CAL, PPD, BIP, and alveolar bone regeneration after 90 days. Owing to the ethical reasons that limited the number of times the dogs could be subjected to general anesthesia, the onset of effects on PD endpoints could not be assessed at the intermediate visits.

The present study suggested that inhibition of cathepsin K may be an effective therapeutic approach to improve clinical care of PD in dogs. In particular, inhibition of cathepsin K by VBX1000 was associated with an improvement in periodontal status regarding gingivitis (BIP) and periodontitis (CAL, PPD, and alveolar bone defect).

The mechanism of action and the relative contribution of cathepsin K activity in the development of gingivitis and periodontitis remains poorly described. Cathepsin K is predominantly secreted by activated osteoclasts, which are the main contributors to bone resorption. Cathepsin K plays an essential role in the bone resorption process by degrading collagens and gelatin, the latter being a product of collagen hydrolysis. In addition, cathepsin K dissolves type I collagen, the major component of the bone matrix (19). Several studies have suggested that cathepsin K likely plays a crucial role in alveolar bone resorption in patients with periodontitis.

In humans, cathepsin K in the gingival crevicular fluid of patients with periodontitis is elevated compared with healthy individuals, reflecting increased osteoclastic activity in periodontal tissues (20, 22, 23, 26, 29, 30). The role of cathepsin K in periodontal bone resorption is supported by studies in animal models of periodontitis showing reduced bone lesion in cathepsin K knockout mice (24), in shRNA-induced cathepsin K-deficient mice (21, 38), and in mice treated with selective cathepsin K inhibitors (25, 27). Together, these studies highlight the predominant role of osteoclast-derived cathepsin K plays in periodontitis. However, cathepsin K in the gingival fluid is produced by osteoclasts, fibroblasts, macrophages, and gingival epithelial cells, contributing to attachment loss and alveolar bone resorption (20). Using a mouse model of bacteria-induced periodontitis, researchers have reported that cathepsin K activity is induced in the gingival connective tissue prior to the establishment of chronic inflammation and osteoclast induction, and they suggested that the early cellular source of cathepsin K may be of fibroblastic origin (39).

In addition to the expected direct effects on bone metabolism, a growing body of evidence supports a regulatory role for cathepsins on the innate and adaptive immune system (28, 40). Cathepsin K has been shown to positively modulate the immune response by favoring activation of Toll-like receptors (TLRs) by pathogen-associated-molecular patterns, such as activation of TLR9 by bacterial CpG DNA. Moreover, pharmacological or genetic inhibition of cathepsin K results in defective TLR9 signaling in dendritic cells stimulated by unmethylated CpG DNA, without affecting their antigen-presenting ability (41). Subsequent *in vitro* experiments in cultured immune cells (macrophages and dendritic cells) have confirmed that knockdown or inhibition of cathepsin K suppresses both TLR9 downstream signaling proteins (24, 27, 38) and autophagy-related proteins (40). In addition, activation of TLR9 by CpG-oligodeoxynucleotides (ODNs) induce cathepsin K expression in isolated gingival fibroblasts (39). In periodontitis models, a major role of TLR9 signaling cascade is strongly supported by studies showing that TLR9 knockout mice are resistant to experimental periodontitis provoked by oral inoculation of *Porphyromonas gingivalis* (42) or by ligature placement (43). Aligned with an interplay between cathepsin K activity and TLR9 activation in mouse models of bacteria-induced periodontitis, bone resorption is consistently associated with increased infiltration of immune cells (macrophages, T cells, and dendritic cells) and increased expression of TLRs (TLR4 and TLR9), autophagy proteins (transcription factor EB [TFEB] and microtubule-associated protein light chain 3 [LC3]) and inflammatory cytokines (interleukin [IL]1β, IL1α, IL6, or tumor necrosis factor alpha [TNFα]) in the periodontium. Both bone resorption and associated inflammation are blunted in cathepsin K knockout mice (24) or are prevented in wild-type mice by silencing cathepsin K (21, 38) or by administering cathepsin K inhibitors (25, 27). In a mouse model of bacteria-induced periodontitis, oral treatment with the prototypical cathepsin K inhibitor, odanacatib, has been reported to decrease the number of macrophages, osteoclasts, TLR4-positive cells, TLR5-positive cells, and TLR9-positive cells in the periodontitis lesion area, as well as reduce levels of certain inflammatory mediators, such as TNFα, IL6, or IL23 (25).

In humans, several small studies assessing the efficacy of bisphosphonate antiresorptive drugs administered orally have yielded favorable results on PD, significantly improving CAL, PPD and alveolar bone loss (44, 45). However, larger studies with longer duration of treatment are still necessary to validate the clinical applicability of bisphosphonate drugs in periodontal treatment. In beagle dogs with moderate-to-severe periodontitis enrolled in a 3-month placebo-controlled trial with alendronate, researchers have reported statistically significant differences in bone mass and bone density between the alendronate and placebo groups; however, they reported a lack of alendronate effects on gingival inflammation and plaque, as well as only a trend toward decreased attachment loss and mobility (46). Finally, the long-term use of bisphosphonates in periodontitis is limited by an increased risk of osteonecrosis of the jawbone in humans (47) and necrosis of the mandibular jawbone matrix in dogs (48, 49). Mechanistically, the antiresorptive activity of bisphosphonates is associated with a strong decrease in bone formation, as assessed via bone turnover markers or the extent of bone forming surfaces in histological sections (50). This decreased bone formation is commonly ascribed to alendronate-induced decreased bone resorption because bone resorption by osteoclasts and bone formation by osteoblasts are connected. In contrast with bisphosphonates, cathepsin K inhibition results in an increased number of osteoclasts; although cathepsin inhibition impairs the ability of osteoclasts to resorb bone matrix, they remain on the bone surface, with the ability to regulate local signaling and maintenance of osteoblast function and activity. Thus, although cathepsin K inhibition effectively limits osteoclast-mediated bone resorption, it may permit preservation of osteoblast function (18). Such a preservation of osteoblast function may explain why inhibition of cathepsin K blocks alveolar bone resorption and promotes alveolar bone healing.

Because the basic principles of the bone resorption process are shared by all mammal species, these results in dogs may be generalizable to humans or other companion animals, but further validation is needed by specific studies.

This PoC study of VBX1000 had several limitations. The present study was a non-blinded, non-placebo-controlled study with a limited number of canine patients. Furthermore, initial scaling and polishing at the inclusion visit may be a confounding factor that may have had favorable effects on gingivitis and periodontitis. In the absence of daily dental care in dogs, however, the bacterial biofilm rapidly reforms after initial dental cleaning. Thus, no improvement of periodontitis could be expected after 90 days of treatment in a placebo group. Because the present study included dogs with stages 2–3 PD, the potential benefits of VBX1000 treatment on higher stages of periodontitis remain to be addressed. Given the apparent safety of VBX1000 use in the two treatment groups throughout the treatment period, the results of this first PoC study in dogs should be confirmed via a larger randomized placebo-controlled, double-blinded study including all stages of PD.

## 5 Conclusion

The results of the present PoC study support the use of a selective cathepsin K inhibitor, such as VBX1000, to improve CAL, PPD, and alveolar bone loss in dogs with mild to moderate PD. With a favorable efficacy to safety ratio, VBX1000 may become a novel drug candidate that should be further validated in canine PD using a larger randomized, double-blinded, placebo-controlled trial.

## Data Availability

The raw data supporting the conclusions of this article will be made available by the authors, without undue reservation.
